# Testing for Divergent Transmission Histories among Cultural Characters: A Study Using Bayesian Phylogenetic Methods and Iranian Tribal Textile Data

**DOI:** 10.1371/journal.pone.0014810

**Published:** 2011-04-29

**Authors:** Luke J. Matthews, Jamie J. Tehrani, Fiona M. Jordan, Mark Collard, Charles L. Nunn

**Affiliations:** 1 Department of Human Evolutionary Biology, Harvard University, Cambridge, Massachusetts, United States of America; 2 Department of Anthropology, Durham University, Durham, United Kingdom; 3 Evolutionary Processes in Language and Culture, Max Planck Institute for Psycholinguistics, Nijmegen, The Netherlands; 4 Human Evolutionary Studies Programme and Department of Archaeology, Simon Fraser University, Burnaby, British Columbia, Canada; 5 Department of Anthropology, University of Missouri, Columbia, Missouri, United States of America; Durham University, United Kingdom

## Abstract

**Background:**

Archaeologists and anthropologists have long recognized that different cultural complexes may have distinct descent histories, but they have lacked analytical techniques capable of easily identifying such incongruence. Here, we show how Bayesian phylogenetic analysis can be used to identify incongruent cultural histories. We employ the approach to investigate Iranian tribal textile traditions.

**Methods:**

We used Bayes factor comparisons in a phylogenetic framework to test two models of cultural evolution: the hierarchically integrated system hypothesis and the multiple coherent units hypothesis. In the hierarchically integrated system hypothesis, a core tradition of characters evolves through descent with modification and characters peripheral to the core are exchanged among contemporaneous populations. In the multiple coherent units hypothesis, a core tradition does not exist. Rather, there are several cultural units consisting of sets of characters that have different histories of descent.

**Results:**

For the Iranian textiles, the Bayesian phylogenetic analyses supported the multiple coherent units hypothesis over the hierarchically integrated system hypothesis. Our analyses suggest that pile-weave designs represent a distinct cultural unit that has a different phylogenetic history compared to other textile characters.

**Conclusions:**

The results from the Iranian textiles are consistent with the available ethnographic evidence, which suggests that the commercial rug market has influenced pile-rug designs but not the techniques or designs incorporated in the other textiles produced by the tribes. We anticipate that Bayesian phylogenetic tests for inferring cultural units will be of great value for researchers interested in studying the evolution of cultural traits including language, behavior, and material culture.

## Introduction

Understanding how cultural phenomena change through time to produce the variation in artifacts, behaviors and institutions seen in the ethnographic and archaeological records is a major challenge. Evolutionary theory and methods have reinvigorated the study of cultural variation by allowing anthropologists and archaeologists to infer the nature of past cultural processes with greater rigor. To this end, the phylogenetic analysis of culture has emerged as a major research approach [1]–[5]. Recent studies have used phylogenetic methods to investigate, for example, the transmission of basketry traditions among Californian Native Americans [6], [7], the spread of prehistoric peoples and technologies [8]–[11], patterns of descent in cultural behaviors among East African societies [12], and the borrowing of linguistic elements in Oceanic [13] and Indo-European languages [14]. Additionally, by modeling historical relationships, phylogenies provide the scaffolding on which to investigate cross-cultural questions involving ancestral states [15], [16], rates of evolution [17], correlated evolution [18], and the occurrence of horizontal transmission [19].

Cultural phenomena can, in principle, diversify through several processes, but to date researchers have focused on two main macro level processes: ‘phylogenesis’ and ‘ethnogenesis’. In phylogenesis, diversification takes place through descent with modification from an ancestral social group, whereas in ethnogenesis it occurs by borrowing and blending of traits among contemporaneous groups [4], [20], [21]. To assess the relative importance of phylogenesis versus ethnogenesis, researchers have employed measures of ‘tree-likeness,’ which quantify the degree to which a set of traits are consistent with a branching-tree model [4]. These measures include the permutation tail probability test (PTP [22]–[24]), the phylogenetic bootstrap [25], the consistency and retention indices [26]–[29], and the network-derived delta index [30], [31]. Simulation studies have shown that standard support measures can be used to infer phylogenesis when these measures are high [32]–[34]. However, these studies have also revealed that such measures are unable to distinguish between ethnogenesis and multiple independent inventions of similar characteristics. Thus, low measures of phylogenetic support are largely uninformative, because they can be due to groups borrowing from each other, or convergent evolutionary change, or a combination of the two [33].

**Figure 1:**
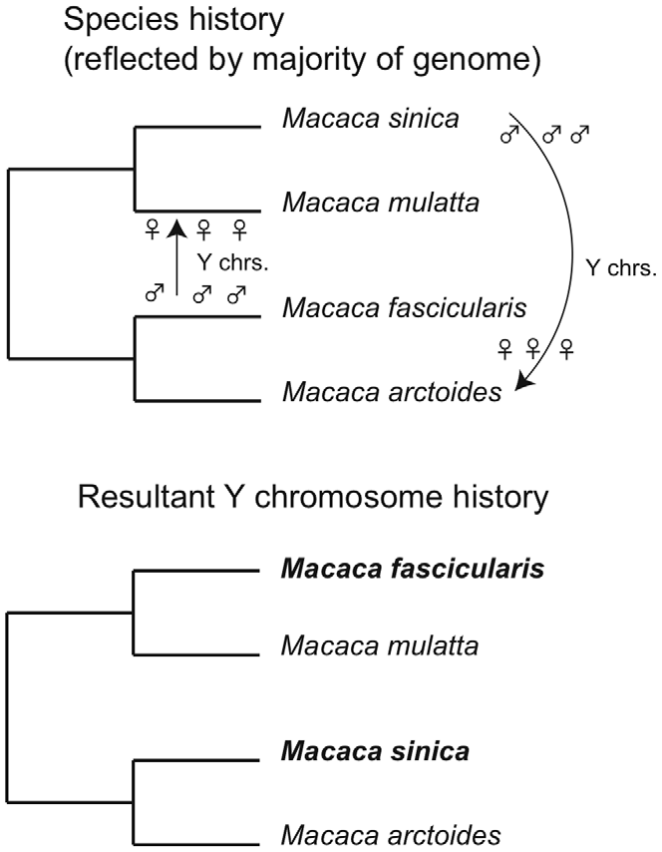
Asymmetric hybridization hypothesis developed by Tosi et al. [**43**] to explain incongruent gene trees in Asian macaque monkeys (genus *Macaca*). **Asymmetric hybridization is shown by arrows that indicate when males of one species breed with females of another.** The male and female hybrid offspring then breed back with the maternal species only. The Y chromosome is a contiguous DNA fragment inherited solely through the paternal lineage. Because of chance processes or female preference, the admixed Y chromosomes become typical of the descendent species, resulting in the bottom phylogeny for Y chromosomes. Note the shifted positions of *Macaca fascicularis* and *Macaca sinica*. This evolutionary process can take multiple generations and involves multiple transmission events. The physical linkage of Y chromosome DNA is the mechanism that produces the transfer of Y chromosomes as a coherent unit and the resultant gene-tree incongruence. Analogously, any mechanism in cultural transmission that produces a necessary linkage of traits during transmission events could result in similar forms of tree incongruence. (modified from [43]).

More generally, it has become desirable to explore methods that can explicitly investigate the processes that produce non-tree-like patterns in cultural data. Contrary to what many archaeologists and anthropologists have assumed [35]–[38], horizontal transmission is not a uniquely cultural phenomenon: it is known to occur in many genetic systems [39]–[42]. An example of how components of an evolving system may become unlinked through time is described in Figure 1. In this case, horizontal transmission produces incongruent gene histories when the males of one species breed with the females of a closely related species (i.e., asymmetric hybridization) [43]. Indeed, descent is strictly tree-like only for a minority of life on Earth, mainly involving sexually reproducing organisms that are separated by substantial amounts of evolutionary time. Biologists have developed approaches to infer horizontal gene transfer, including network techniques, tests for the appropriateness of a tree model, and methods to detect gene-tree incongruence [5], [42], [44]. Thus, the horizontal transfer of cultural ideas and practices is not necessarily an intractable problem for cultural phylogenetics [45].

In this paper, we use Bayesian methods of phylogenetic reconstruction to address two models of cultural evolution that have been widely discussed in the literature [5], [46]–[48]. These models – the ‘hierarchically integrated system’ model and the ‘many coherent units’ model [46] – draw from the concepts of ethnogenesis and phylogenesis in populations. Rather than considering the histories of individual traits, however, the models are concerned with understanding the transmission dynamics of sets of traits. Compared to studies that treat traits as independent, these models have received less empirical scrutiny.

The hierarchically integrated system model proposes that cultural assemblages are composed of two types of characters: those belonging to a core tradition that evolves through phylogenesis, and peripheral characters that are commonly exchanged among groups and can be gained or lost with relative ease. Bayesian phylogenetic analysis is a useful way to investigate this model because it allows researchers to classify characters into separate partitions (e.g. ‘core traits’ and ‘peripheral traits’) and then to test if allowing rates of change to vary between partitions provides a better model for the evolution of the data than assuming equal rates of change. Because peripheral characters change through horizontal transfers between extant groups as well as through cultural innovation, they are expected to exhibit different rates of change from core characters that evolve by innovation alone.

The many coherent units model proposes that cultural assemblages consist of multiple groups of characters that have different transmission histories. These groups are analogous to sections of a chromosome that are sufficiently close that they tend to transfer together during sexual reproduction, rather than being broken up by genetic recombination. In the cultural case, correlated transmission may arise because the traits are functionally or symbolically interrelated (e.g. the rituals, texts and institutions of a religion), or because they are repeatedly borrowed from the same source (e.g. French words in the English language). This model is testable in a Bayesian framework because, unlike in a parsimony analysis, different trees can be incorporated into the analysis as independent parameters [49], [50].

**Figure 2:**
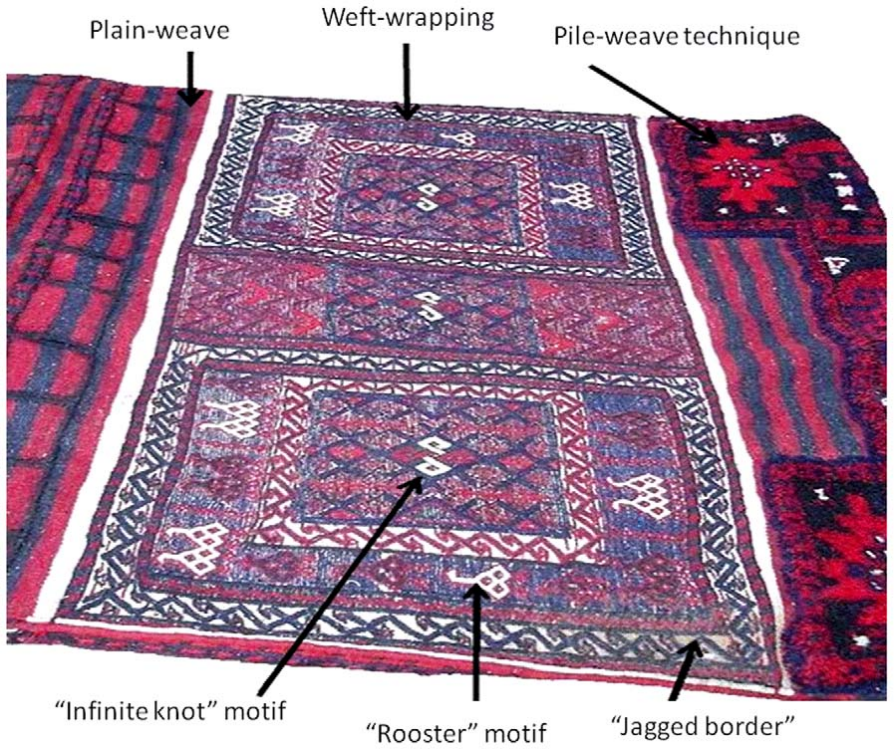
Section of a Bakhtiari saddle-bag illustrating examples of the technical and decorative traits used in the analyses.

We tested predictions from these two models using data derived from Iranian tribal textiles that were collected by Tehrani and Collard [21] (Figure 2). Tehrani and Collard's [21] ethnographic research showed that the majority of techniques and designs used by weavers were acquired ‘vertically’ in two contexts: on an individual level from their mothers, and at a community level from ancestral populations. Weavers have few opportunities to learn traits from members of other tribes due to endogamous marriage practices and social norms that restrict the ability of women to travel far from their camp or village. However, Tehrani and Collard [21] noted that one class of traits was more likely to circulate among groups. These traits comprise the designs that are woven into pile carpets (‘pile-weave designs’), which are often copied from cartoons provided by urban rug merchants and/or learned through temporary employment in commercial workshops.

We used the textile data to test the two hypotheses described above. In terms of the hierarchically integrated system hypothesis, weaving techniques and ‘flat-weave designs’ represent a plausible core tradition, since they are relatively isolated from outside influences. Pile-weave designs, on the other hand, might be expected to comprise peripheral elements that are adopted and discarded according to market demands. We therefore predicted different rates of evolution for pile-weave design characters, as they would be more affected by horizontal transfer. Empirical [4] and simulation [33] studies have shown that independent ethnogenetic transfers can increase estimated evolutionary rates if they produce patterns consistent with homoplasy (character state similarity not due to vertical descent). However, horizontal transfers can also decrease the evolutionary rates inferred from comparative data, for example when the ancestral state transfers to a lineage with a derived character state (i.e., homoplasy is potentially obscured). This effect has been demonstrated in some simulation studies, where systematic transfer among historically related societies has tended to erase independent changes that would have been reconstructed in the absence of horizontal transmission, thereby biasing estimates of evolutionary rates downward for traits with greater horizontal transfer [32].

To explore the effects of horizontal transfer on inferred evolutionary rates in the present context, we simulated character evolution and transfer on the most parsimonious tree obtained by Tehrani and Collard [21]. We compared the inferred evolutionary rates of the simulated characters with and without horizontal transfers to assess whether the transfers increased or decreased the rates. We then used the simulation results to develop a directional prediction regarding the effect of horizontal transfers on evolutionary rates within the hierarchically integrated system hypothesis.

Alternatively, the textile data might fit the many coherent units hypothesis. Market trade could have caused the pile-weave design characters to become a coherent cultural component with a transmission history that differs from the other textile characters. Unlike the hierarchically integrated system model, the many coherent units hypothesis does not predict that pile-weave design characters have different rates of evolution than other kinds of characters. Rather, the many coherent units hypothesis predicts that the pile-weave design characters produce a tree topology that differs from the tree topology yielded by the other textile characters.

## Materials and methods

### 2.1 Data

The data for this study are textile design and construction characteristics recorded by JJT from museum collections and during 6 months of ethnographic fieldwork in Iran between May 2001 and June 2003 (see [21] for a breakdown of sources). A total of 122 characters were derived from the textile sample (Table S1). They included 42 techniques of preparation and fabrication (e.g. spinning and knotting techniques), 56 flat-weave designs and 24 pile-weave designs (for examples see Figure 2). The characters were coded as presence/absence in a binary matrix that reflects the presence of characters used by a particular tribe in any of their textiles. That is, for a character to be coded as present for a tribe, the tribe was observed to use the character in at least some of its textiles. Characters coded as absent for a tribe were not observed in any of the tribe's textiles.

Following Tehrani and Collard [21] we used an archaeological textile assemblage—the Pazryk collection—as an outgroup to infer the likely ancestral states of the textile characters in our analyses. The Pazryk collection was recovered from ice-filled tombs of a nomadic population that inhabited the Altai Mountains of Siberia 2400-2300 years ago [51]. The age and quality of preservation of these textiles provide the best available information on the historical roots of weaving among Central and Western Asian nomadic pastoralists [52].

### 2.2 Simulation of horizontal transfers within a hierarchically integrated system

The hierarchically integrated system hypothesis predicts that rates of evolution should differ for the pile-weave design characters versus the technique and flat-weave design characters. To establish whether the rates for the pile-weave design characters would be expected to be higher or lower than the non-pile characters we carried out a set of character simulations.

**Figure 3:**
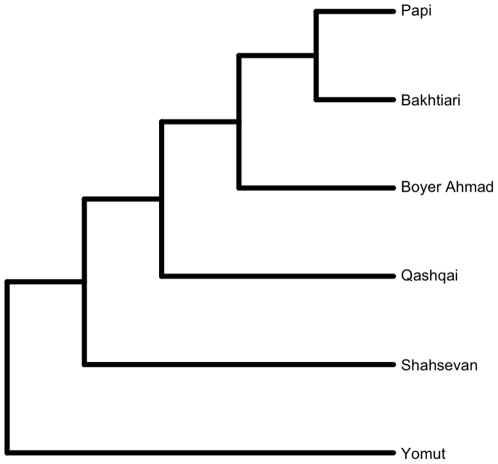
The Grafen transformation of Tehrani and Collard's [**21**] parsimony tree of the textile data. Character evolution was simulated along the branches of the tree and independent horizontal transfers of individual characters were simulated at the tips.

We simulated traits on a Grafen transformation [53] of the parsimony tree topology (no branch lengths) inferred previously from the same data set [21]. The simulation process required branch lengths that are roughly proportional to time, which means using an ultrametric tree in our case because all the tribes exist in the present day. The Grafen transformation is a standard way to generate an ultrametric tree in the absence of good temporal information. It sets the age of each node equal to one less than the number of descendant taxa (Figure 3). We did not use the branch lengths from our Bayesian analysis (described below) because this would have introduced circularity into the simulations.

We simulated the evolution of 100 characters, each with an instantaneous transition rate of 0.123. This transition rate was the median transition rate of the empirical dataset when optimized via maximum likelihood on the Grafen transformed tree. For these calculations, we used functions fitDiscrete and simchar in the R package ‘geiger’ [54]). We did not allow for any horizontal exchange of these 100 characters, making them analogous to our hypothesized evolutionary process for the non-pile-weave design characters.

Under the hierarchically integrated system hypothesis, pile-weave design characters are peripheral elements that should exhibit a different rate of evolution from the non-pile-weave design characters that belong to the core tradition. To create comparable simulated pile-weave design character sets, we generated 100 sets of 30 characters under three horizontal transfer processes. Under the local borrowing condition, each tribe had a 30% chance of adopting the character state of one of its sister tribes on the tree. Sister tribes were those separated by only one internal node. We also conducted an anti-local borrowing condition in which each tribe had a 30% chance of adopting the character state of any tribe separated by two internal nodes. Under anti-local borrowing, sister tribes never borrowed character states directly from one another. Lastly, we simulated a global borrowing condition in which each tribe had a 30% chance of adopting a character state from any of the other tribes on the tree. All transfers occurred among the terminal taxa after vertical evolution along the tree topology. This simulation process is similar to that of Greenhill et al. [32].

We eliminated characters that were invariant, because such characters are not typically included in cultural and morphological data sets for phylogenetic analysis. After eliminating the invariant characters, we were left with simulated datasets of 25 to 30 characters to compare with the 100 characters that experienced no horizontal transfer, which is comparable to our empirical dataset of 24 pile-weave design characters and 98 non-pile-weave design characters. We then used the same maximum likelihood estimator (function fitDiscrete in ‘geiger’ [54]) to infer the rates of evolution of the characters, which was repeated for each of the three types of horizontal transfer. We assessed the effect of each form of horizontal transfer on the median rate of evolution by comparing the simulated sets of pile-weave design characters to the 100 simulated non-pile-weave design characters that did not experience any horizontal transfer.

### 2.3 Bayesian phylogenetic inference

Bayesian phylogenetic inference proceeds by assessing consecutive ‘proposals’ of combinations of a dataset and a model of evolution. The model consists of a number of parameters, the most basic being: a tree topology, a set of branch lengths and an evolutionary model for character change. The latter is modeled as the probability of instantaneous change between character states, e.g. from 0 to 1 and 1 to 0. Branch lengths are proportional to the amount of evolutionary change occurring along them. A likelihood score for each character is then calculated, based on the changes that must take place in order to observe the distribution of that character's states on the proposed topology and branch lengths.

After calculating the likelihood of each character given a particular model proposal, the likelihoods for all characters are combined to obtain the likelihood score for a single proposal of a tree and parameter values. The parameters and likelihood score are recorded, and the process is repeated in the next iteration. The iterations take place through a Markov Chain Monte-Carlo (MCMC) process. The MCMC then explores the likelihood landscape by adopting new parameter values in a search that favors parameters that give a higher likelihood. This distribution of trees samples the topologies and branch lengths such that phylogenies with higher support are sampled to a greater extent. The investigator can summarize this posterior distribution by producing a consensus tree of the highest-frequency clades and mean branch lengths in the sample, with nodes annotated with their clade credibility value (i.e., the probability that the node appears in the posterior sample).

### 2.4 Initial model exploration

We used MrBayes b3.1.2 [55], [56] to infer phylogenetic trees. During model selection, we used the harmonic mean of the MCMC chain to determine the model with the highest marginal likelihood [50]. We assessed harmonic means after an empirically determined burn-in period. For subsequent analyses, we termed the model best supported by the data the ‘base model’.

We modeled the transition rates in each textile character between 0 (absent) and 1 (present). Our first parameter characterized whether rates of gain (0 to 1) and loss (1 to 0) were equal, which enabled us to test whether our data were best described by symmetric or asymmetric transition rates. Our second parameter characterized the amount of rate variation across all characters—that is, whether some textile characters evolved faster than others, or if rates were similar across the set of characters. Although our data consisted of presence-absence codes, we tested for rate asymmetry by coding the data as ‘standard’ rather than as binary. We did this because binary data are interpreted by MrBayes as analogous to genetic ‘restriction sites.’ The restriction site model in MrBayes is a direct application of a model for rate asymmetry in DNA data. Known as the F81 model, this model uses character state frequency to derive one invariant rate asymmetry for all characters. This assumed invariance of rate asymmetry across characters is unrealistic for anatomical characters because, unlike DNA, no single underlying mechanism causes the asymmetry [57]. Similarly, we had little reason to think a single mechanism produces transition asymmetry for design motifs, weaving techniques, or even that the asymmetry for different design motifs should be invariant. Lewis [57] suggested solving the analogous problem for anatomical data by drawing rate asymmetries from a Beta distribution. This invokes the same number of new parameters as the F81 model, but allows for variance in asymmetry across sites. We considered this model to be more realistic for our data, and we implemented it as the symmetric Dirichlet hyperprior for ‘standard’ data in MrBayes.

We used a ‘gamma parameter’ to test for variation in evolutionary rate across sites. This parameter does not adjust the rate asymmetry for sites. Rather, it adjusts all rates for a site by a multiplier that allows for rate heterogeneity. We used a standard setting that approximates (for computational efficiency) the gamma parameter value by fitting four discrete rate categories. Like anatomical data sets used in phylogenetic studies, cultural data sets exhibit a bias in the types of characters coded. Specifically, characters are only included in a dataset if they have been observed in at least one taxon in the sample. This corresponds to the MrBayes code ‘noabsencesites’, in which no single character can have an absent state for all taxa. MrBayes modifies its likelihood equation to account for this bias.

These model parameters were not simply imposed on the analysis, but were tested statistically with a likelihood score to assess whether the additional parameters are justifiable on statistical grounds. Bayesian analyses do not always favor more complicated models, because simpler models can actually achieve higher marginal likelihoods [58]. Under an initial assumption that treats all models as equally probable (‘flat priors’), our posterior belief in one model over the other model is reflected by differences in the harmonic mean likelihoods generated by each model [50].

To search the parameter space efficiently, we used multiple MCMC chains per run. We ran three ‘hot chains’ that proposed large parameter changes in order to explore parameter space more expansively. A single ‘cold’ sampling chain periodically adopted the hot chain states and continuously recorded the states of the chain. Large sampling intervals are usually required to reduce autocorrelation between states in the chain, but here the small number of taxa allowed us to sample trees (i.e. record the parameter values and tree topology) every 100 generations. We conducted six such MCMC runs of 100,000-iterations for each analysis.

The length of the burn-in period was determined empirically such that results obtained prior to the likelihood reaching stationarity were discarded. We took the final 900 trees of the post-burn-in from each of six chains to compile the posterior distribution of 5400 trees per analysis. From this distribution, we constructed a consensus phylogenetic tree and assessed how it compared to the bootstrapped parsimony tree inferred by Tehrani and Collard [21].

### 2.5 Hypothesis testing

#### 2.5.1 The hierarchically integrated system model

After determining through simulation whether horizontal transfers would increase or decrease evolutionary rates, we tested the appropriate prediction through two analyses. First, we modified the base model by partitioning the data set into the pile-weave and non-pile-weave design characters. We then unlinked the rate parameter for each partition and re-ran the MCMC analyses. Unlinking a parameter across partitions allows it to take on different values for each partition, while constraining the other parameters to be the same across all characters. We assessed the support for the partitioned model relative to the base model with a Bayes factor comparison based on the harmonic means of the model likelihoods. The harmonic mean is a standard approximation of the marginal likelihood, the latter being required for Bayes factor analysis [59]. Unlike the frequentist approach, which rejects a null hypothesis, Bayes factors represent a summary of the odds for one model over another. Based on Kass and Raftery's [58] logarithmic scale for interpretation, Bayes factor values between 0 and 2 are barely worth mentioning, values between 2 and 5 represent positive evidence, values between 5 and 10 are strong evidence, and values greater than 10 constitute very strong evidence.

Second, we tested for a difference in rates by examining the results for our gamma model for character evolution (see above). The gamma model allows characters to have different rates of evolution, and the posterior sample of these rates produces a unique rate for each character. We used our simulations to justify our prediction of higher or lower rates for the characters with more horizontal transfer. We then compared the inferred rates of the pile-weave design characters (hypothesized to have more horizontal transfer) to the rates of the non-pile-weave design characters (hypothesized to have less horizontal transfer) with a Mann-Whitney U test.

#### 2.5.2 The many coherent units model

The second hypothesis predicts that a partitioned Bayesian analysis should support different phylogenies for pile-weave design characters as compared to the non-pile-weave design characters that include both flat-weave designs and weaving techniques. To test this, we allowed different classes of traits to produce different evolutionary histories, that is, we unlinked the topologies across partitions of the data. This method of unlinking topologies for *a priori* partitions has precedence in genetic studies that have investigated topological incongruence due to different descent histories of different genes [49], [50], [60]–[63]. For example, Suchard et al. [49] unlinked topology between partitions and used Bayes factors to estimate model support in order to infer the horizontal transmission of viral types among HIV patients. As Gray et al. [5] have suggested, this approach should also be appropriate for studying cultural traits that are potentially learned and transmitted in different ways.

By unlinking tree topology during Bayesian tree inference, each partition was allowed to have an independent tree. MrBayes recorded the trees for both partitions during each sampled generation. We calculated the marginal likelihoods of the post-burn-in posterior distribution for each partition and used Mesquite [64] to generate the two consensus trees. If patterns of descent in pile-woven designs differ from other textile traits, we predict positive Bayes factor support for the topologically partitioned model, as compared to the base model.

The primary empirical and simulation support for this statistical test comes from Galtier and Daubin [63], who showed that a maximum likelihood (ML) difference metric that is similar to the Bayes factor exhibited more evidence for multiple gene trees in bacteria than in metazoa. This result was consistent with theoretical predictions given the facility with which some bacteria share particular genes across species lineages. Galtier and Daubin [63] also obtained consistently high ML differences for simulated gene evolution on completely unlinked gene-trees. Thus, both their empirical and simulation results indicate that the ML difference reliably detected topological incongruence.

## Results

### 3.1 Initial model exploration

**Figure 4:**
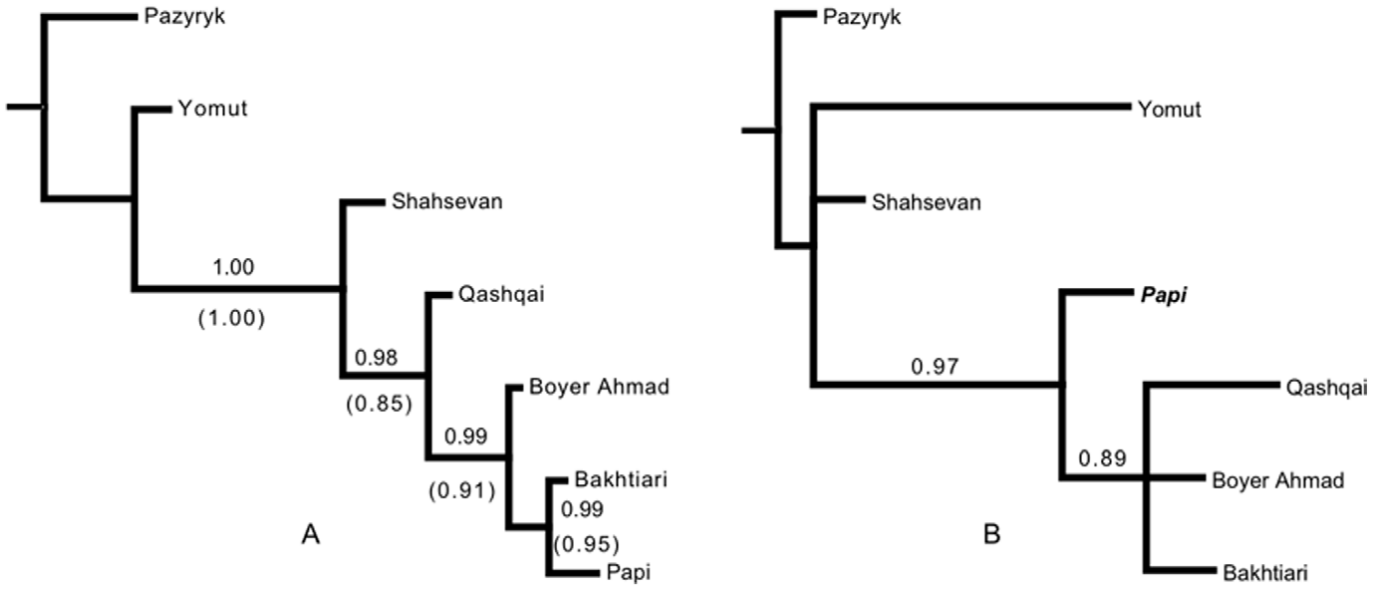
Consensus trees from the Bayesian phylogenetic analysis of Iranian textile characters. Numbers at nodes show clade credibility values, which reflect the proportion of trees in the posterior probability sample that share a given node. Panel A shows the tree inferred from all characters (credibility values outside parentheses) and from non-pile-weave design characters (credibility values inside parentheses) using the base model. Panel B shows the tree inferred from pile-weave design characters using the base model. Note the shifted position of the Papi.

A simple model with a symmetric rate and no cross-site rate variation yielded the best posterior probability as reflected by the harmonic mean likelihoods across the MCMC chains (Table 1). Support for the simple model was positive when compared with a model that added the gamma parameter. A model without any rate asymmetry was preferred over all other models, each Bayes factor for comparison comprising ‘very strong evidence’ under Kass and Raftery's [58] categories. Model fit was worsened by the inclusion of either rate asymmetry or a gamma parameter (Tables 1 and 2). We therefore used the simple model with a symmetric rate of character change and without gamma as the base model. Using the base model and with the complete data set, our final posterior distribution comprised a sample of 5400 trees. This set of trees produced a highly resolved consensus topology (Figure 4a).

**Table 1:** Harmonic means of log likelihoods (lnL) for different evolutionary models.

model	lnL
symmetric transition rate (S)	−509.36
symmetric transition rate + gamma (SG)	−509.96
asymmetric transition rate (A)	−544.76
asymmetric transition rate + gamma (AG)	−543.03

**Table 2:** Bayes factor comparison of evolutionary models described in Table 1.

	S	SG	A
SG	1.20	-	-
A	70.80	69.60	-
AG	67.34	66.14	−3.46

Note: Bayes factors were calculated as 2*(column harmonic mean *ln* likelihood - row harmonic mean *ln* likelihood). Positive Bayes factors indicate support for the model in the columns across the top, negative values for the model in rows to the left. S: symmetric transition rates without gamma, SG: symmetric transition rates and gamma, A: asymmetric transition rates without gamma, AG: asymmetric transition rates and gamma.

### 3.2 Hypothesis testing

#### 3.2.1 Hierarchically integrated system model

**Figure 5:**
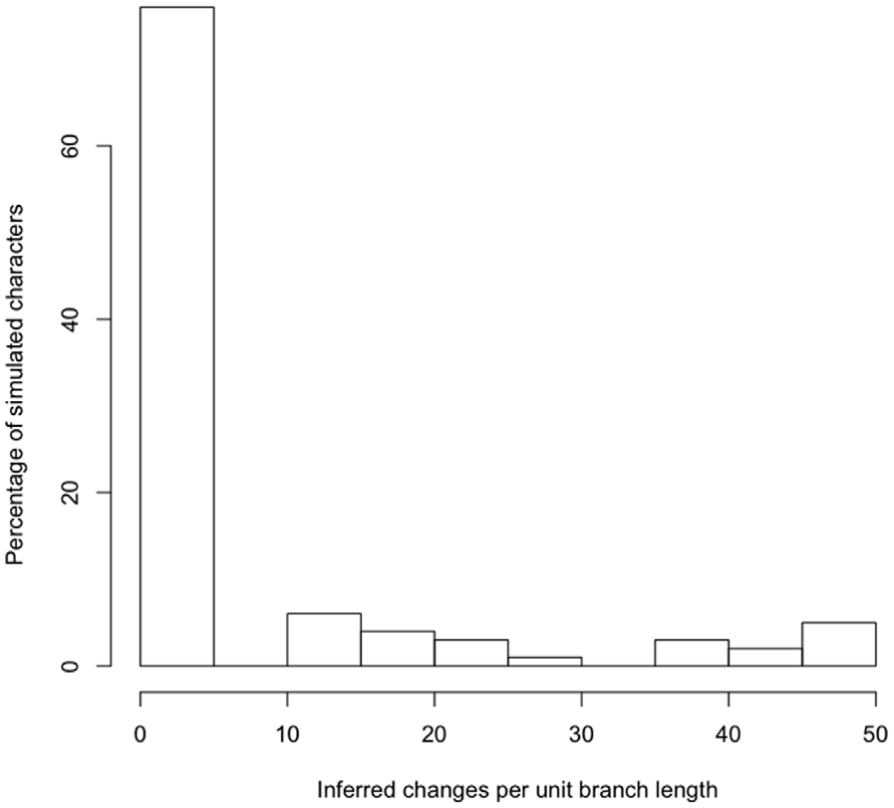
Histogram of inferred rates of evolution for 100 characters simulated without horizontal transfers. This non-normal distribution also was characteristic of the rates inferred under the horizontal transfer conditions.

The simulation experiments indicated that horizontal transfers increased inferred evolutionary rates on the Grafen transformed tree topology (Figure 3). The characters were all generated with a rate of 0.123 changes per unit branch length. The median inferred rate for the 100 characters simulated without any horizontal transfer was 0.19, but the mean was a highly divergent 7.04. The distribution of inferred rates is highly non-normal (Figure 5), so the median is the preferred measure of central tendency in this case.

We simulated 100 sets of 30 characters that all experienced local independent horizontal transfers (transfers among sister taxa). Of these simulations, 97% exhibited higher median rates of evolution than in the characters without horizontal transfer. We also conducted 100 simulations of 30 characters each that experienced anti-local transfers among taxa separated by 2 internal nodes on the phylogeny. Under this condition, 98% of simulations exhibited greater median rates than did the characters without horizontal transfers. Lastly, we simulated 100 sets of 30 characters each that experienced global transfers that were equally probable among any of the taxa. Under global transfers, 94% of the simulations exhibited greater median rates than the median rate for characters without horizontal transfers.

Given the simulation results, we predicted that pile-weave design characters would exhibit higher median evolutionary rates than the other textile characters if they had experienced more independent horizontal transfers as peripheral elements of a hierarchically integrated system. This prediction was not supported by the model with partitioned transition rates for pile-weave and non-pile-weave design characters. Allowing different rates for each character partition slightly worsened the harmonic mean likelihood compared to the base model (−510.13 versus −509.36, Bayes factor = 1.48 in favor of the base model).

Furthermore, the analyses revealed no support for the gamma model, which allows for rate variation over the base model without gamma (Table 2). Within this gamma model, however, we found significant support for a small difference in the median rate of character evolution (Mann-Whitney U test, p = 0.03, median rate of change per unit branch length for non-pile-weave design characters = 0.997, median rate change per unit branch length for pile-weave design characters = 1.000).

#### 3.2.2 Many coherent units model

Unlinking the tree topology for the pile-weave and non-pile-weave design characters produced a substantially improved likelihood and positive Bayes factor support (10.44 in favor of different topologies, ‘very strong evidence’). The consensus tree from the non-pile-weave design characters had the same topology as the consensus tree inferred from the complete data set, and clade credibility values were all equal to or greater than 0.85 (Figure 4a). The pile-weave design characters produced a less resolved topology for some nodes, but for one node they supported a different topology than the non-pile-weave design characters (Figure 4b). This node puts the Papi in a basal position relative to the Boyer Ahmad, Bakhtiari, and Qashqa'i. The latter three tribes are linked in a monophyletic clade with credibility support of 0.89. We also found positive Bayes factor support for this node by comparing the inferred topology shown in Figure 4b to a topology that constrained the Papi as sister to the Bakhtiari for the tree of pile-weave design characters (5.88 in favor of the inferred topology). These findings suggest that the pile-weave design characters have a different descent history compared to the non-pile-weave design characters. This difference can be seen in Figure 4 and is consistent with the hypothesis that these traits comprise a cultural component that was borrowed by some or all of these groups from a non-ancestral source.

## Discussion

Using Bayesian phylogenetic approaches, we inferred independent evolutionary histories for two sets of Iranian textile characters, enabling us to test models about the underlying processes of culture change. The simple base model inferred a robustly supported consensus tree that matched the consensus bootstrap parsimony tree obtained previously from these data [21]. We also obtained the same tree, with similar clade credibility values, from less favored, more complex models (unpublished results).

The analyses provided very limited support for the idea that a history of commercial trade produced a different rate of inter-tribe transmission of individual pile-weave design characters. Based on our simulations, such a process should have resulted in a greater median transition rate for pile-weave design characters in the gamma model. While we found significant support for the predicted increased rate, the amount of rate increase was minimal (an increase of 0.003 changes per unit branch length compared to the overall rate). The small magnitude of increase is probably why Bayes factors did not support either the partitioned model or the gamma model, both of which allowed for rate variation. Because the magnitude is small, it is of little consequence to the likelihood of the data. Horizontal transfers may occur more frequently in the pile-weave design characters, but the increased rate is extremely small and has little impact on the distribution of character states among the tribes.

We note that it is also conceivable that horizontal transfers would increase the variance of inferred evolutionary rates without affecting their central tendency. This effect was difficult to assess with our particular simulations given the boundary conditions of the maximum likelihood estimation procedure. The empirical data, however, showed no support for different variances of the pile-weave design and other textile characters (F-test, ratio of variances = 1.04, p = 0.96, numerator df = 97, denominator df = 23; nonparametric Fligner-Killeen test, median χ^2^ = 0.15, p = 0.70, df = 1). These findings are consistent with Tehrani and Collard's [21] cladistic analyses of the textile traits, which found no significant differences in the retention indices of pile and non-pile characters. We can therefore conclude that there is little evidence to suggest that Iranian tribal weaving traditions evolve in line with the “hierarchically integrated system” model.

In contrast, the results of the analyses are strongly consistent with the multiple coherent units model. This model proposes that pile-weave design characters transfer as a group and do so separately from the other characters—a process that produces separate transmission histories. Consistent with the coherent units model, we found positive support for different topologies for non-pile-weave design and pile-weave design characters. The clade credibility values for these trees are reduced (Figure 4), but this is understandable given the concomitant reduction in the number of characters used to infer each tree. A single clear topological disagreement is manifest in the comparison of the non-pile-weave and pile-weave trees: that being the position of the Papi textiles relative to the Boyer Ahmad, Bakhtiari, and Qashqa'i.

A caveat about the Bayes factor test for multiple tree topologies exists on mathematical grounds [50], [61]. When the model for character evolution on a single tree is overly simple compared to the actual process of character evolution, the Bayes factor test for different trees is thought to produce spurious positive results due to model misspecification. Positive results for multiple trees may be suspect when the underlying data are fit best by the most complex character model available, as this might indicate that the character model is insufficiently complex to describe how the characters truly evolved. In our study, however, the simplest model for character evolution was favored in the model exploration for a single tree topology. So, the caveat does not apply.

Two potential explanations may account for the topological difference between the best-fit phylogeny for the pile-weave design characters, and the best-fit phylogeny for the other characters. One is that the Boyer Ahmad, Bakhtiari, and Qashqa'i adopted pile-weave design characters from a common external source, leaving the Papi in a basal position. The other is that the Papi adopted pile-weave designs from the Yomut and/or Shahesevan, which would have the effect of shifting the Papi to a more basal position. Given that the Papi currently live hundreds of miles away from the Shahsevan and Yomut (∼300 km and ∼800 km, respectively, over deserts and mountainous terrain) and there is no evidence that the Papi were ever neighbors of the Shahsevan or Yomut, the second scenario seems unlikely. In contrast, the first scenario is consistent with ethnographic and historical data. To reiterate, the main media for the introduction of pile designs from foreign sources—workshops and cartoons—are both linked to commercial rug production. Commercial rug production has a long history among the Qashqa'i, Bakhtiari and Boyer Ahmad. For example, pile rugs attributed to the Qashqa'i were being traded in urban and export markets as early as the mid-eighteenth century [52], [65]. A distinctive feature of these groups' commercial weavings is the extent to which they imitate well-known urban and courtly designs. For example, the Bakhtiari ‘kheshti’ (brick) pattern appears to be based on the classical ‘four garden’ design, which was popularized during the Safavid Dynasty (1507–1732). Other common imitations of urban designs include the so-called Herati pattern, medallion ornaments and Shirazi prayer rugs [66].

We suggest that the topology of the pile-weave design tree reflects the involvement of the Qashqa'i, Boyer Ahmad and Bakhtiari in commercial textile markets, such that they each adopted pile-weave designs from an external source common to all three. This transfer would have facilitated the spread of commercially popular tribal and urban designs. Because women belonging to different tribes would have been competing within a single regional market, they would be expected to adopt the designs that were most popular among consumers. Design popularity and their physical co-occurrence on design cartoons may be the mechanism that produced the package-like transfer of these traits and resulted in the observed topological differences.

This explanation is consistent with the exclusion of the Papi from the clade linking the pile designs of the Bakhtiari, Qashqa'i and Boyer Ahmad. The available historical evidence suggests that the Papi began commercial production much later than the other three tribes, compared to whom they were both geographically and politically remote. Lacking a coherent centralized leadership structure, the Papi were much less integrated into the political economy of Iran than the Qashqa'i, Bakhtiari and Boyer Ahmad. The leaders of the latter groups, the ‘khans’, were major players on the national stage, with the power to levy taxes and raise armies. They provided an important cultural and economic link between ordinary tribe members and wider Iranian society. In the case of rug weaving, the khans actively encouraged commercial production as a means of increasing tax revenues, and even set up their own workshops that were managed by their wives [67]. So-called ‘bibibaff’ rugs (‘woven by ladies’) were specifically produced for urban consumers and aristocrats, and are today valuable antiques [68]. The absence of comparable institutions among the Papi might explain why they relied more on their own traditional patterns, rather than borrowing from outside the tribe.

### Conclusions

Our study highlights a new approach for investigating a fundamental question in cultural transmission and evolution: Do cultural traits exhibit different histories of transmission? If so, can assemblages be characterized as “hierarchically integrated systems” comprising “core” and “peripheral” traits, or as “multiple coherent units”? While both these models have been widely discussed [5], [46]–[48], few techniques have been developed to infer them from comparative ethnographic and archeological data. Our study demonstrates that Bayesian phylogenetic inference provides a statistically rigorous framework to investigate these possibilities.

Our analyses of Iranian tribal textile assemblages found that the transmission histories of pile-weave design characters differ from other textile characters. They do not, however, represent a collection of peripheral traits that move freely between the branches of a single “core” phylogeny. Instead, it appears that the textile characters comprise two distinct and phylogenetically coherent packages. Crucially, this kind of analysis cannot be easily carried out with the parsimony methods used in previous studies of material culture evolution [6]–[10], [21]. This is because, unlike the harmonic mean likelihood, parsimony statistics such as the retention index can only be used in reference to a single topology. Thus, our Bayesian approach advances this field by rendering open to scientific inquiry a hypothesis that was previously untestable with the sort of comparative data used in this study. We anticipate that this approach will be useful for many other types of cultural data, including language, behavior, and material culture.

## Supporting Information

Table S1 Character list.
(0.13 MB DOC)
