# Supplementary material for: Testing for Divergent Transmission Histories among Cultural Characters: A Study Using Bayesian Phylogenetic Methods and Iranian Tribal Textile Data
Source: PLoS One. 2011 Apr 29;6(4):e14810. doi: 10.1371/journal.pone.0014810 (PMC3084691; doi:10.1371/journal.pone.0014810)
Supplement: Table S1 — Character list. (0.13 MB DOC) [file pone.0014810.s001.doc]

SUPPORTING TABLE 1. Character list

| **Character** | **Trait** | **Category** |
| --- | --- | --- |
| **1** | ground loom | technique |
| **2** | vertical loom | technique |
| **3** | open shed | technique |
| **4** | needle weave | technique |
| **5** | perforated cards | technique |
| **6** | spinning | technique |
| **7** | plying | technique |
| **8** | rolled felts | technique |
| **9** | shaped felts | technique |
| **10** | weft-twining (1) | technique |
| **11** | weft twining (2) | technique |
| **12** | twined baskets | technique |
| **13** | colied baskets | technique |
| **14** | weft-wrapping | technique |
| **15** | countered wefts | technique |
| **16** | diagonal wefts | technique |
| **17** | weft-faced plain | technique |
| **18** | goat-hair weft weave | technique |
| **19** | slit-tapestry | technique |
| **20** | shared warp weave | technique |
| **21** | interlocking warps | technique |
| **22** | warp-faced plain weave | technique |
| **23** | double-faced warp pattern | technique |
| **24** | wapr-faced *jajim* weave | technique |
| **25** | weft-knotting | technique |
| **26** | weft-wrapped knots | technique |
| **27** | pile knots | technique |
| **28** | symmetrical knots | technique |
| **29** | asymmetric knots | technique |
| **30** | depressed warp threads | technique |
| **31** | weft shots | technique |
| **32** | 1 weft shot | technique |
| **33** | 2 weft shots | technique |
| **34** | 3 weft shots | technique |
| **35** | gabbeh weave | technique |
| **36** | plaiting | technique |
| **37** | weft float brocade | technique |
| **38** | compound weave with pile floats | technique |
| **39** | goat hair selvages | technique |
| **40** | corded selvages | technique |
| **41** | ends folded | technique |
| **42** | ends stitched | technique |
| **43** | A-shapes on bands | flat-weave design |
| **44** | X-shapes on bands | flat-weave design |
| **45** | Evil eye | flat-weave design |
| **46** | Bricks in alternating colours | flat-weave design |
| **47** | Central medallion | flat-weave design |
| **48** | piano-key pattern | flat-weave design |
| **49** | all-over patterns | flat-weave design |
| **50** | diamond lattice | flat-weave design |
| **51** | diamond dazzle pattern | flat-weave design |
| **52** | stepped diamond | flat-weave design |
| **53** | comb-edge diamond | flat-weave design |
| **54** | diamond chain | flat-weave design |
| **55** | hooked diamonds | flat-weave design |
| **56** | memling | flat-weave design |
| **57** | animal-head hooks | flat-weave design |
| **58** | rose bush | flat-weave design |
| **59** | stylised shrubs | flat-weave design |
| **60** | angular flowers | flat-weave design |
| **61** | dart-like flowers | flat-weave design |
| **62** | floating darts | flat-weave design |
| **63** | peacock motif | flat-weave design |
| **64** | simple animal shapes | flat-weave design |
| **65** | 2-headed birds | flat-weave design |
| **66** | simple human shapes | flat-weave design |
| **67** | rosettes | flat-weave design |
| **68** | palmettes | flat-weave design |
| **69** | serrated palmette | flat-weave design |
| **70** | serrated palmette with trunk & branches | flat-weave design |
| **71** | ram-horn diamond | flat-weave design |
| **72** | *Gul* | flat-weave design |
| **73** | 8-pointed stars | flat-weave design |
| **74** | stepped star | flat-weave design |
| **75** | beetle | flat-weave design |
| **76** | oak leaf | flat-weave design |
| **77** | geometric leaves | flat-weave design |
| **78** | triangle boat border | flat-weave design |
| **79** | saw-tooth border | flat-weave design |
| **80** | crab-claw border | flat-weave design |
| **81** | interlocking hooks | flat-weave design |
| **82** | bird hooks | flat-weave design |
| **83** | animal head hooks | flat-weave design |
| **84** | helmet-like hooks | flat-weave design |
| **85** | soldat | flat-weave design |
| **86** | V border | flat-weave design |
| **87** | S borders | flat-weave design |
| **88** | continuous S's | flat-weave design |
| **89** | interlocking s's | flat-weave design |
| **90** | lozenge s's | flat-weave design |
| **91** | geometric swaztika | flat-weave design |
| **92** | reciprocal triangles | flat-weave design |
| **93** | reciprocal laleh | flat-weave design |
| **94** | reciprocal rose border | flat-weave design |
| **95** | diamond with projecting arrows | flat-weave design |
| **96** | infinity motif constructed from ram horn | flat-weave design |
| **97** | endless knots | flat-weave design |
| **98** | diamond border | flat-weave design |
| **99** | ornaments repeated on plain field | pile-weave design |
| **100** | diamond medallion | pile-weave design |
| **101** | plain field with animal shapes | pile-weave design |
| **102** | field divided into compartments | pile-weave design |
| **103** | khesthi compartments | pile-weave design |
| **104** | ensi style | pile-weave design |
| **105** | lions | pile-weave design |
| **106** | naturalistic palmette | pile-weave design |
| **107** | tree of life in rosettes | pile-weave design |
| **108** | tree of life in palmettes | pile-weave design |
| **109** | ram-horn tree | pile-weave design |
| **110** | *kochak* | pile-weave design |
| **111** | *chemche* | pile-weave design |
| **112** | naturalistic cypress trees | pile-weave design |
| **113** | abstract cypress | pile-weave design |
| **114** | *boteh* | pile-weave design |
| **115** | large inverted boteh | pile-weave design |
| **116** | *Tekke Gul* | pile-weave design |
| **117** | serrated leaves | pile-weave design |
| **118** | serrated leave boat border | pile-weave design |
| **119** | curled leaf boat border | pile-weave design |
| **120** | herati pattern | pile-weave design |
| **121** | *Yomut gul* | pile-weave design |
| **122** | interlocking bird swaztika | pile-weave design |
